# Open access and article processing charges

**DOI:** 10.1186/1747-597X-1-19

**Published:** 2006-07-17

**Authors:** Stephan Arndt

**Affiliations:** 1 Department of Psychiatry, Carver College of Medicine, University of Iowa, Iowa City, IA 52242, USA; 2 Department of Biostatistics, College of Public Health, University of Iowa, Iowa City, IA 52242, USA; 3 Iowa Consortium for Substance Abuse Research and Evaluation, Iowa City, IA 52242, USA

## Abstract

*Substance Abuse*, *Treatment*, *Prevention*, *and Policy *uses a medium that provides the broadest possible worldwide readership. Anyone in the world can read articles without charge. Since the articles published here will hopefully help inform policy this is exactly the right target – free and unlimited access. Beginning July 2006, article-processing charges will be applied to papers published in this journal.

## Background

*Substance Abuse*, *Treatment*, *Prevention*, *and Policy *uses a medium that provides the broadest possible worldwide readership. Articles can be freely read by anyone in the world without charge. Since the articles published here will hopefully help inform policy this is exactly the right target.

The traditional strategy for disseminating work has been to publish in paper journals that charge the readers directly or through society memberships. Typically, academicians subscribe to a few journals within their own specialized area. Articles within those journals are often aimed to a narrow audience and consequently, the articles are not widely read outside the particular field. Open access is an alternative.

## The open access model

Material in open access journals is equally available to anyone, anywhere free of charge at the time of publication. This fact alone is a huge advantage for this journal and for the authors. Since we aim to affect policy, the work here absolutely has to cross over and breakdown boundaries. Readers have access to this journal regardless of the financial resources of their region, libraries, or universities. Furthermore, common web searches such as through Google or Yahoo will point to relevant and freely accessible papers within the journal.

There are other advantages. Authors retain the copyright for their own work. Articles are indexed in PubMed immediately on publication [1]. Furthermore, manuscripts are deposited in a number of safe open access archives, including PubMed Central [2], INIST (France) [3], Potsdam University (Germany) [4] and Koninklijke Bibliotheek (The Netherlands) [5].

As with traditional publishing, open access publishing has underlying costs. To continue to fund open access publication, from July 2006, an article-processing charge (APC) will be levied on articles accepted for publication. Submitted articles that are not accepted will incur no charge.

Article processing charges pay for developing and maintaining electronic tools for peer review and publication, preparation in various formats for online publication, securing inclusion in PubMed as soon as possible after publication, securing full text inclusion in a number of permanent archives such as PubMed Central, securing inclusion in CrossRef (enabling electronic citation in other journals that are available electronically), and of course for immediate world-wide barrier-free open access to the full text.

The APC will be covered in full or part if authors come from one of BioMed Central's member institutes [6], and many funding agencies explicitly allow for coverage of open access publication in research grant proposals [7]. Notably, in the USA grants from the National Institutes of Health allow such costs.

The Editors will consider applications for waivers of the APC on a case-by-case basis, and we will do our best to support authors in instances of genuine financial hardship. We understand the APC may be steep for an out of pocket expense.

Work of the Editors, editorial board and peer-reviewers is voluntary and unpaid. We have, however, sought funding to ensure we can support a number of APC waivers. The Iowa Consortium for Substance Abuse Research and Evaluation [8] has donated 4000 USD to this cause, and we continue to search for other donor organizations, although always with the integrity of our editorial independence in mind.

Authors, readers, and editorial board members can suggest external funding sources to help grant waivers. The foundation might be from regional or otherwise specialized focus (e.g., alcohol prevention), and we can try to accommodate that. Please send your suggestions to the editor.

## Conclusion

We are committed to furthering open access in the field of substance abuse, to reach the widest audience possible, and to inform policy. We hope that you will support this cause by submitting your next article to an open access journal.

## Competing interests

The author(s) declare that they have no competing interests.
